# Engineering the Signal Resolution of a Paper-Based Cell-Free Glutamine Biosensor with Genetic Engineering, Metabolic Engineering, and Process Optimization

**DOI:** 10.3390/s24103073

**Published:** 2024-05-12

**Authors:** Tyler J. Free, Joseph P. Talley, Chad D. Hyer, Catherine J. Miller, Joel S. Griffitts, Bradley C. Bundy

**Affiliations:** 1 Department of Chemical Engineering, Brigham Young University, Provo, UT 84602, USA; 2 Department of Microbiology and Molecular Biology, Brigham Young University, Provo, UT 84602, USA

**Keywords:** biosensor, glutamine, cell-free protein synthesis (CFPS), cancer

## Abstract

Specialized cancer treatments have the potential to exploit glutamine dependence to increase patient survival rates. Glutamine diagnostics capable of tracking a patient’s response to treatment would enable a personalized treatment dosage to optimize the tradeoff between treatment success and dangerous side effects. Current clinical glutamine testing requires sophisticated and expensive lab-based tests, which are not broadly available on a frequent, individualized basis. To address the need for a low-cost, portable glutamine diagnostic, this work engineers a cell-free glutamine biosensor to overcome assay background and signal-to-noise limitations evident in previously reported studies. The findings from this work culminate in the development of a shelf-stable, paper-based, colorimetric glutamine test with a high signal strength and a high signal-to-background ratio for dramatically improved signal resolution. While the engineered glutamine test is important progress towards improving the management of cancer and other health conditions, this work also expands the assay development field of the promising cell-free biosensing platform, which can facilitate the low-cost detection of a broad variety of target molecules with high clinical value.

## 1. Introduction

Diagnostic medical tests provide critical support for the healthcare industry. In 2019, there were an estimated 1 billion office-based physician visits in the US [1], and the Centers for Disease Control and Prevention estimates that 70% of medical care decisions depend on test results [2]. An estimated 4 billion medical laboratory tests are ordered each year to provide critical information for optimal disease management and increased survival rates for many health conditions, including cancer, the second-leading cause of death in the US [3,4,5,6,7].

Glutamine is widely known to play a crucial role in cancer growth and proliferation by providing nitrogen for amino acid biosynthesis, as well as carbon to replenish the TCA cycle [8,9,10,11,12,13,14]. Not surprisingly, glutamine metabolism is upregulated in several cancers [15]. This has led to the development of various cancer therapies that aim to restrict cancer cells’ glutamine supply [15,16,17,18,19]. One such treatment, CB-839, inhibits glutaminase, a key enzyme that breaks down glutamine [20,21,22]. However, glutamine metabolism in healthy cells must remain appreciably balanced to prevent dangerous side effects, where the digestive system is particularly vulnerable [20,23,24,25,26]. Clinical trials have been initiated to target the delicate glutamine metabolism, where mixed results indicate that further research is needed to fully elucidate successful treatment-management strategies and the role of glutamine as a biomarker [22,27,28]. Frequent testing of patient glutamine levels could enable patients to achieve and maintain the appropriate treatment dosage to optimize the tradeoff between successful cancer treatment and dangerous side effects.

Conventional medical lab tests for glutamine, such as high-performance liquid chromatography (HPLC), have limited portability, high costs, and slow result times, which restrict patient access to treatment-monitoring tests [29,30]. This has motivated efforts to develop alternative glutamine biosensors utilizing a variety of biochemical and analytical methods [31,32,33,34,35,36,37]. Lim et al. developed the Q-SHINE assay, in which a split glutamine-binding protein recombines with a split reporter protein that dimerizes upon binding of glutamine to produce a signal [31]. This exciting study presents a unique and effective way to detect glutamine, though this is a relatively slow assay that requires several hours to obtain results, and the assay has not been demonstrated in a portable form. Lam et al. engineered a glutamine sensor using a different glutamine-binding protein labelled with acrylodan and a reference fluorophore [34]. The glutamine concentrations are correlated to the change in modulation frequency. Tanigawa et al. harnessed cellular nutrient-response mechanisms to create an in vitro glutamine sensor, discovering novel protein–glutamine-binding interactions [36]. While these and other novel methods to detect glutamine [31,32,33,34,35,36,37] represent important progress in the field of glutamine sensing, no commercialized, portable glutamine tests are available. Thus, patients suffering from cancer and other diseases do not have laboratory equipment nor expertise to conduct frequent testing.

We previously reported a glutamine sensor using “cell-free” or in vitro protein synthesis, a technology capable of low-cost, shelf-stable, portable, non-invasive, and rapid biosensors [38,39]. The prior work had critical assay background limitations. Thus, the rationale for the work presented here is to overcome these limitations. To achieve this goal, we engineer an innovative cell strain and compare the individual and combined capabilities of metabolic engineering, genetic engineering, and process optimization to improve the signal resolution of the cell-free glutamine sensor.

The open environment of the cell-free protein synthesis system enables direct mixing of biosensor reagents with a sample for biomarker interaction and detection [40,41,42,43,44]. Biosensing cell-free reactions typically produce a reporter protein, such as superfolder green fluorescent protein (sfGFP) or β-galactosidase (β-gal) [45,46]. All 20 proteinogenic amino acids are required for protein translation, and recent work utilized this dependence to engineer a biosensor for amino acids [47]. The biosensor formulation lacks the amino acid analyte, while each of the other 19 amino acids are supplied in excess. The biosensor is mixed with a sample, where the limiting reactant in protein translation is the amino acid analyte in the sample. While this method worked well to detect most of the 20 amino acids, reporter protein production was unhindered in the absence of exogenous asparagine, aspartate, glutamate, and glutamine [47]. The supply of missing amino acids was largely attributed to metabolic enzymes present in the crude cell extract [47]. In the case of glutamine sensing, this false-positive limitation was partially overcome by engineering the sensor with methionine sulfoximine (MSO) to inhibit glutamine synthetase [38]. However, the assay readable window and signal resolution were limited by a glutamine assay background that caused false positive results [39].

Cell-free protein synthesis is also useful for NMR protein studies, where the residual metabolic conversions of one amino acid into another cause isotope label scrambling [48,49]. This problem has inspired significant work to understand and overcome the same metabolic pathways that interfere with the cell-free glutamine assay application. Small-molecule inhibitors and combinatorial gene knockouts have been used to target many major interconversion pathways with success [50]. Additionally, glutamine released by proteolytic digestion of plentiful lysate proteins is also mitigated in NMR work with protease inhibitor cocktails [50]. To reduce metabolic interference in cell-free NMR studies, Bernhard et al. engineered a specialized mutant of the A19 strain with targeted gene knockouts to facilitate cell-free protein synthesis with very little amino acid interconversion [50,51].

While the specially modified A19 strain could likely facilitate glutamine biosensing with a very low assay background, a BL21 Star DE3 strain confers the following advantages for our low-cost, rapid, colorimetric biosensor application. (1) The A19 strain does not produce the prolific T7 RNA polymerase, which must be supplied from a separately purified mixture for cell-free protein synthesis [50], while the T7 RNA polymerase can be readily produced by BL21 Star DE3 during cell extract preparation upon induction with isopropyl β-D-1-thiogalactopyranoside (IPTG) to eliminate a separate source of purified protein, thereby streamlining production and reducing cost. (2) BL21 Star DE3 is capable of faster growth rates and biomass accumulation, which is likely to yield a faster sensor response [52]. (3) BL21 Star DE3 has a deficiency of the lon and OmpT proteases [53,54,55], which provides a dual benefit to our sensing application to theoretically (a) improve biosensor signals by reducing reporter protein degradation and (b) decrease background glutamine production from protease activity. (4) BL21 Star DE3 carries the RNase mutation rne131, which significantly decreases RNase activity and thereby increases mRNA half-life, which facilitates higher translation capacity and faster sensor signal response [56]. Thus, in this work, we created BL21 Star DE3 Δ*lac* Δ*glnA*, where gene knockouts disable β-galactosidase and glutamine synthetase, respectively. The Δ*lac* mutation removes endogenous β-gal in the cell extract source that would otherwise cause a false positive when the β-gal reporter is used in the colorimetric sensor. β-gal produced by translation during a sensing reaction is facilitated by a template plasmid.

Small molecule enzyme inhibitors that target the activity of undesired metabolic enzymes and proteases can be readily added to the open reaction environment of cell-free biosensors [57]. Our prior work to enable glutamine biosensing demonstrates that small molecule inhibitors can effectively reduce glutamine assay background [38], and prior NMR studies have also reported that other inhibitors can reduce activity of other metabolic enzymes [49]. Inhibitors are a particularly useful approach to inhibit enzymes that are essential to cellular viability. Efforts to knock out such enzymes can prove cytotoxic, while inhibitors can be strategically used and optimized to transiently block pathways during various cellular processes to obtain a similar effect to a gene knockout [58]. In the context of glutamine biosensors, we have previously demonstrated the effectiveness of methionine sulfoximine (MSO), a competitive inhibitor of the primary pathway for glutamine synthesis, in lowering glutamine background in a cell-free protein synthesis biosensor.

There is a cost tradeoff of inhibitors due to their single-use preparation [59]. The cell-free assay reagents cost ~USD 0.40 per 0.1 mL of assay reagents [60]. MSO (purchased in 5 g quantity and added at 80 mM) costs ~USD 0.30 per 0.1 mL of assay reagents while protease inhibitor costs ~USD 0.05 per 0.1 mL of assay reagents. Thus, ~40% of the previously reported glutamine assay cost is MSO. Additionally, the efficacy and specificity of inhibitors can vary, where some inhibitors may only exhibit partial inhibition of the desired pathway [61]. Furthermore, many inhibitors exhibit off-target effects on other enzymes [62], potentially impacting protein synthesis and biosensor signal strength. As such, the use of inhibitors in cell-free protein synthesis requires careful selection and optimization to be effective.

## 2. Materials and Methods

### 2.1. Cell-Free Protein Synthesis

*E. coli* cell extract for cell-free protein synthesis reactions was prepared according to previously reported protocols [38,39,63], except in this work we used BL21-Star™ DE3 (Invitrogen, Carlsbad, CA, USA) cells, which we engineered in this work with Δ*lac* and Δ*glnA* frameshift mutations (Figure S6). All growth media were 2 × YT media supplemented with 1 g/L glutamine unless otherwise noted. Cells were induced with IPTG to a final concentration of 1 mM when the optical density (OD600) reached 0.5, and cells were harvested when the OD600 was approximately 3.

Cell extract was prepared by sonication as reported previously [64]. Cells were resuspended in 10 mL per gram of wet cells in Buffer A [64] and centrifuged at 6000 relative centrifugal force (RCF) units for 15 min. The supernatant was then discarded. After washing three times, the cells were resuspended in 1 mL of Buffer A per gram of wet cells. A 15 mL falcon tube containing the cell suspension was placed on ice. The sonication lysis was performed with a Vibra-cell VCX 400 probe sonicator with a CV 26 probe (tip diameter of 3 mm; Sonics and Materials, Newtown, CT, USA) at a tip frequency of 20 kHz at 15% amplitude. Twelve cycles of sonication consisting of a 2 min pulse followed by a 5-to-15 min rest period were used to prevent overheating. After sonication, the resulting lysate was centrifuged at 12,000 RCF for 30 min. The supernatant was collected and separated into multiple equivalent aliquots. One aliquot of 2 mL was dialyzed at 4 °C against 1 L of Buffer A with stir bar agitation for 16 h, and the dialyzing solution was discarded and replaced by 0.7 L of fresh Buffer A and incubated at 4 °C for 24 h. The dialyzed lysate was removed from the membrane chamber and centrifuged at 12,000 RCF for 5 min to remove the visible precipitant material. For both the dialyzed and undialyzed cell extract aliquots, equivalent fractions were separated for treatment with 4× MSO (final concentration of 320 mM in the cell extract), or 4× protease inhibitor (EDTA-free Pierce^TM^ Protease Inhibitor Tablets, ThermoScientific (Waltham, MA, USA)), or both. A runoff reaction was conducted for 30 min at 37 °C and 280 RPM for each cell extract condition.

The PANOxSP cell-free protein synthesis reactions were prepared as previously reported [38,39]. Glutamine was omitted from the PANOxSP preparation and was added separately only where explicitly noted.

DNA plasmids were used for expression of either sfGFP or β-galactosidase as indicated. The chlorophenol red-β-D-galactopyranoside (CRPG) (Gold Bio, St. Louis, MO, USA) substrate changes color from yellow to purple when the catalyst β-galactosidase is present. Cell-free reactions contained 12 nM of plasmid, 0.6 mg/mL of CRPG (for β-gal reactions), 25% (*v*/*v*) cell extract, and PANOxSP (concentrations listed above), with the balance of the reaction volume being water. Cell-free protein synthesis reactions producing sfGFP were conducted in 30 μL liquid reaction volumes in 2 mL microcentrifuge tubes. Reactions were incubated at 37 °C, 280 RPM for 1 h and subsequently measured with the Biotek (Winooski, VT, USA) SynergyMx plate reader.

### 2.2. Paper-Based Colorimetric Cell-Free Protein Synthesis

Paper-based, lyophilized β-galactosidase reactions were prepared with chromatography paper (Thick Chromatography Paper Grade 238, VWR 28342-036, VWR International, Radnor, PA, USA) as previously described [39]. To conduct the paper-based assay, the respective batches of lyophilized, paper-based cell-free protein synthesis reactions were randomly assigned into triplicates, and 12 μL of aqueous glutamine solutions of the indicated concentrations were added as samples to initiate the cell-free protein synthesis biosensing reactions. The reactions were promptly covered with transparent tape and placed in an incubator at 37 °C, and test papers were photographed (sample image shown in Figure S5) with a Samsung smartphone every 3 min for 144 min. The image at t = 63 min was used twice, once at t = 63 min and at t = 60 min due to technical difficulties that prevented image capture at t = 60 min.

Images were cropped with Adobe Lightroom and repeat images of each test paper were arranged in rows on Microsoft PowerPoint version 16. No modifications were made to the original images except for cropping. A sample original image is included in the Supplementary Materials. Quantitative image analysis in CIELAB color space was conducted as previously described [39]. The quantitative color change of a reaction at each time point was normalized relative to the first and last photo in each row, except for test papers 2000 (-) for each condition, and 20 µM, 2 µM, and 0 µM (Figure S3), which did not exhibit a substantial color change. These were normalized to saturated color changes for adjacent tests that changed color to purple.

### 2.3. Multiplex Automated Genome Engineering (MAGE)

Multiplex automated genome engineering (MAGE) chromosomal modifications were conducted based on previously published protocols [65,66,67], with the following specifications and modifications. The novel sequence for the pJG1219 MAGE plasmid (Figure S6) contains a medium-copy origin of replication (p15A ORI) and AmpR as an ampicillin-resistance marker. The plasmid is constructed to contain the gene for the cymR repressor protein under control of a constitutive promoter, while the genes to express the lambda red beta protein and mutL_E32K protein are placed under the Pcym promoter. Thus, the genes necessary for successful MAGE oligo incorporation can be induced with IPBA.

BL21 Star DE3 cells were first transformed by electroporation with the pJG1219 plasmid. Briefly, BL21 Star DE3 cells were grown in an overnight 5 mL culture. Further, 1.5 mL of the resultant growth media were centrifuged for 1 min at 14,000 RPM to pellet the cells, after which the supernatant media was discarded. The cells were washed three times, each time with 0.5 mL of 5% glycerol, which was added to the pellet for resuspension and spun down again. After three washes, the cell pellet was then suspended in 40 μL of 5% glycerol. Then, 2 μL of miniprep pJG1219 plasmid were added to the cell suspension and mixed gently with a pipette. The cells were added to an electroporation cuvette, and the cells were electroporated with a brief pulse. Within 10 s after electroporation, 0.5 mL of LB media was immediately added to the cuvette and gently mixed. The media were aspirated and incubated in a microcentrifuge tube for 1 h at 37 °C, shaking at 280 RPM. The recovery suspension was then diluted serially and plated onto LB ampicillin plates to isolate individual colonies containing the pJG1219 plasmid.

The MAGE mutagenesis was conducted based on previously published protocols, with the following specifications [65,66,67]. BL21 Star DE3 cells transformed with the pJG1219 plasmid were grown overnight (8 to 10 h) in 5 mL LB ampicillin culture tubes. Then, 0.5 mL of overnight culture was transferred to 40 mL of autoclaved media and incubated at 37 °C, 280 RPM. When the OD reached 0.2, the cells were induced with 80 μL of 50 mM IPBA (500×, in ethanol solvent). When the OD reached 0.6 to 0.8, the cells were promptly chilled on ice. Cells were pelleted by centrifugation at 14,000 RPM and the supernatant media was discarded. The cells were washed three times, each time with 0.5 mL of 5% glycerol that was added to the pellet for resuspension and spun down again. After three washes, the cell pellet was then resuspended in 40 μL of 5% glycerol. Further, 2 μL of 100 μM single-stranded DNA oligos (designed as stated above) were added to the cell suspension and gently mixed with a pipette. The cells were added to an electroporation cuvette, and the cells were electroporated with a brief pulse. Within 10 s after electroporation, 0.5 mL of LB sterile media was immediately added to the cuvette and gently mixed. The media were aspirated and incubated in a microcentrifuge tube for 4 h at 37 °C, shaking at 280 RPM. This extended recovery time is critical to allow for multiple cell-replication cycles to ensure that some of the daughter cells become homozygous for the mutation.

The recovery suspension was then diluted serially and plated onto LB ampicillin plates to isolate individual colonies. We observed that after 4 h of recovery, 100 μL of media diluted by a factor of 100, 1000, or 10,000 could be used to isolate single colonies. We have observed that between 1% and 20% of the colonies contained the desired mutation. This process was conducted in two stages, first with oligos to knock out the *lacZ* gene, and after successfully isolating a colony containing the desired mutation and having retained the pJG1219 plasmid, MAGE was repeated with this cell line but to now incorporate oligos targeting the *glnA* gene. The Δ*lac* colonies were screened using X-gal plates. 

For the Δ*glnA* frameshift mutation, colonies were screened phenotypically by patching colonies on both minimal media plates and minimal media plates supplemented with glutamine. The cells’ failure to grow on the minimal media plates without glutamine supplementation confirms phenotypic Δ*glnA* knockout. Furthermore, the auxotroph cells successfully grew on LB ampicillin plates, but to a notably smaller size for a particular growth period.

A further qualitative phenotypic growth study with and without glutamine supplemented media was conducted with liquid media. An equivalent inoculum volume of a cell suspension of BL21 Star DE3 *E. coli* was added to each of 5 mL test tubes with varying liquid media types and glutamine-supplementation levels. The test tubes were incubated at 37 °C for 12 h and periodically photographed to observe time-dependent turbidity trends. Glutamine was supplemented as one initial bolus to achieve the indicated mg/mL concentration. Negative control culture tubes received no inoculum of cells.

## 3. Results and Discussion

In this work, we engineer the signal resolution of a paper-based, cell-free glutamine biosensor with genetic engineering, metabolic engineering, and process optimization to overcome previously encountered assay background limitations [39]. The assay background mitigation strategies are studied both individually and in systematic combinations to reveal a quantitative tradeoff between assay signal strength and signal-to-noise ratio. These results are used to select an optimal biosensor formulation to demonstrate the engineered signal resolution in a paper-based colorimetric biosensor.

**Figure 1:**
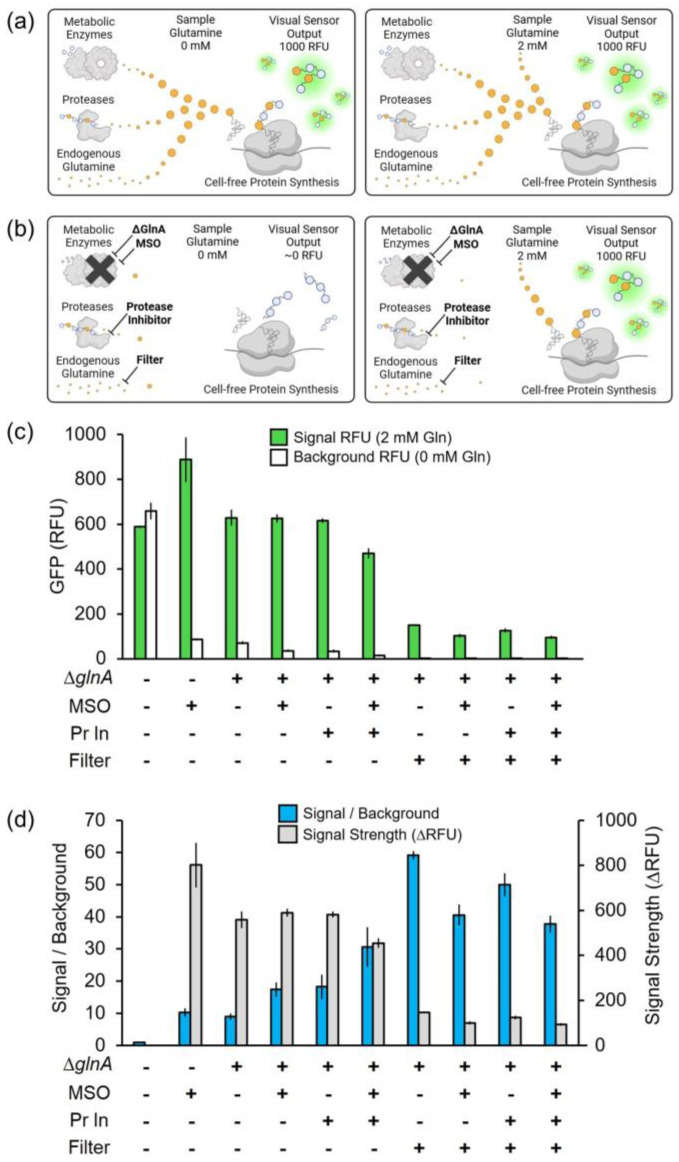
Overview and comparison of cell-free glutamine assay background sources and mitigation strategies. Metabolic enzymes, proteases, and endogenous glutamine are sources of glutamine in crude lysate cell-free protein synthesis reactions, which produce the sfGFP reporter protein (**a**) independent of sample glutamine concentration or (**b**) proportional to sample glutamine concentration as a result of assay background mitigation. (**c**) The sfGFP fluorescence for biosensing reactions initiated with and without glutamine is compared for different background-mitigation strategies. (**d**) Assay signal-to-background ratio and signal strength were computed from the fluorescence values in (**c**). The assay background-mitigation approaches include a glutamine synthetase knockout (Δ*glnA*), methionine sulfoximine (MSO), protease inhibitor (Pr In), and cell extract filtration. Error bars represent one standard deviation of the three replicates.

This work presents a quantitative analysis (Figure 1) of four key strategies to reduce glutamine assay background, including the individual and combinatorial effects of the *glnA* knockout mutation, MSO, protease inhibitor, and filtration by dialysis membrane.

### 3.1. GlnA Knockout

A recent NMR study reported that a *glnA* knockout mutation eliminated cell-free protein synthesis background production in the absence of glutamine [50]. The NMR cell-free protein synthesis work was performed with an *E. coli* K12 A19 strain. To streamline the production of cell extract for our biosensor application, we performed a *glnA* knockout in a specialized BL21 Star DE3 Δ*lac* strain, which produces prolific T7 RNA polymerase in situ during extract preparation. The BL21 Star DE3 Δ*lac* Δ*glnA* specialized strain for biosensing glutamine was created with frameshift mutations to the *lacZ* and *glnA* genes using oligo-mediated multiplex automated genome engineering (MAGE) [65,66,67,68] (Figure S6).

**Figure 2:**
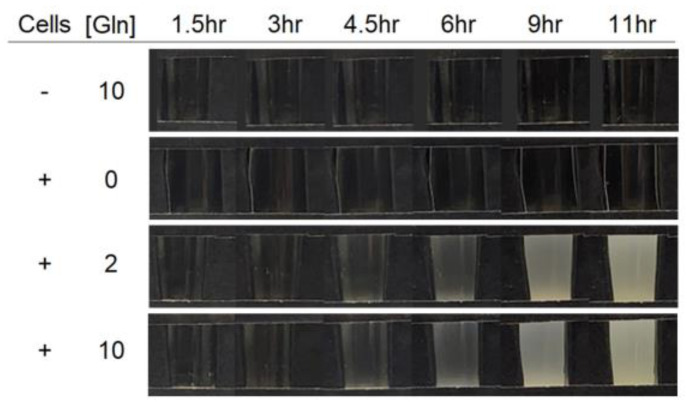
Phenotypic cell growth photographs of the *E. coli* BL21 Star DE3 Δ*lac* Δ*glnA* glutamine auxotroph presented in this work. An equivalent inoculum volume of a cell suspension was added to fresh minimal media in 5 mL culture test tubes supplemented with glutamine, as indicated by the concentration, which is provided in mg/mL. The test tubes were incubated at 37 °C for 12 hr and periodically photographed in front of a dark background to observe time-dependent turbidity. [Gln] indicates the concentration of glutamine in mM. The (-) indicates a culture tube that received no inoculum of cells.

The auxotroph BL21 Star DE3 Δ*lac* Δ*glnA* cell phenotype was confirmed by liquid culture growth in minimal media, with and without glutamine supplementation (Figure 2). The cells in minimal media supplemented with glutamine grew to a qualitatively observable extent, while in the same period of time, the media that were not supplemented facilitated no observable growth.

**Figure 3:**
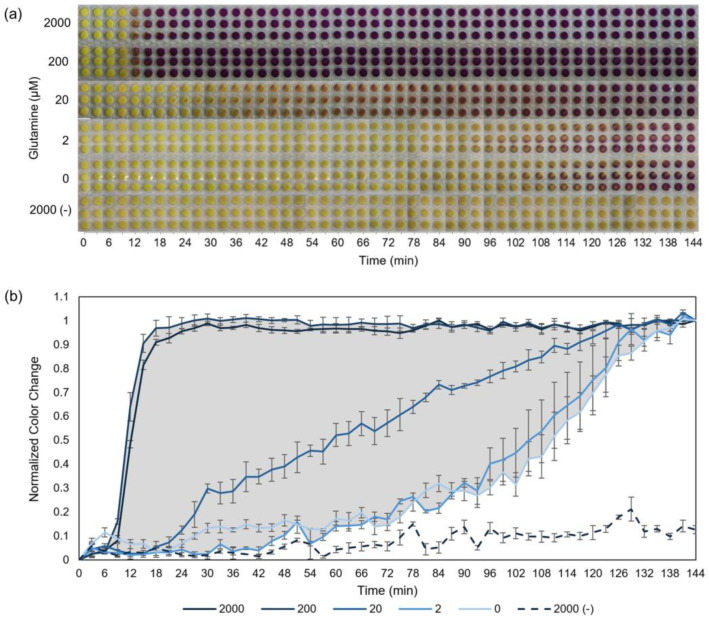
Paper-based glutamine sensor made with Δ*glnA* cell extract, MSO, and protease inhibitor. (**a**) Sequential photographs of the colorimetric, lyophilized, paper-based glutamine sensors produced using the assay improvements presented in this work. Cell-free β-galactosidase (β-gal) synthesis reactions were prepared with the yellow substrate chlorophenol red β-d galactopyranoside (CRPG), which is converted into a purple product by β-gal. Glutamine was omitted from the test formulations, which were pipetted onto paper discs, flash frozen, and lyophilized. Paper tests were hydrated with 12 μL of either 0, 2, 20, 200, or 2000 μM aqueous glutamine samples, as indicated on the vertical axis. Cell extracts were prepared with our BL21 Star DE3 Δ*lac* Δ*glnA* cells, and purified β-gal plasmid DNA was added to the reactions in all cases except for the negative controls, listed as 2000 (-), which were prepared in parallel without any plasmid but with 2000 μM glutamine. (**b**) Quantitative color change derived from (**a**). The signal resolution is represented by the shaded region, which depicts the difference between the assay background and saturated signal. Error bars represent one standard deviation of the three replicates.

A glutamine biosensor produced with cell extract made from the standard BL21 Star DE3 strain has a trivial signal-to-background ratio of 1 (Figure 1c,d). A cell-free glutamine biosensor made with the BL21 Star DE3 Δ*lac* Δ*glnA* cell extract produces an appropriate sfGFP signal response to 2 mM glutamine, and the low background response associated with 0 mM glutamine results in a signal-to-background ratio of 9, which is similar to the ratio enabled by the addition of MSO (Figure 1c,d). Neglecting the initial cost of creating a Δ*glnA* knockout strain, the genetic engineering can enable similar assay signal-to-background performance while reducing the assay cost by ~40%. As described in later sections of this report, additives further reduce assay background in Δ*glnA* cell extract. This work focuses on enhancing the signal resolution of the biosensor rather than focusing on achieving the lowest possible sensor cost, motivating the formulation combining genetic engineering and additives, which is shown in Figure 3.

### 3.2. Methionine Sulfoximine (MSO)

As previously reported, MSO effectively reduces the assay background of a cell-free glutamine biosensor from standard BL21 Star DE3 extract by inhibiting glutamine synthetase present in clarified cell lysate [38]. Importantly, the assay signal is not adversely affected by MSO. This work provides a quantitative comparison that the addition of MSO improves the signal-to-background from the trivial ratio of 1 to a ratio of 10 by purportedly inhibiting glutamine synthetase (Figure 1c,d). Interestingly, within the context of extract made from Δ*glnA* cells, the signal-to-background ratio further increases from 9 to 17 upon MSO addition (Figure 1c,d). If glutamine synthetase was the only enzyme that MSO interacts with we would expect no additive benefit to the signal-to-background ratio. This finding suggests that MSO impacts other glutamine generation pathways, possibly glutaminase or other transaminases. This is not completely unexpected as the best A19 strains for glutamine labeling for NMR studies included additional knockouts *asnA*, *ansA*, *ansB*, *glnA*, *aspC*, and *ilvE* [50]. Furthermore, within the context of Δ*glnA* cell extract treated with protease inhibitor cocktail, adding MSO further increases the signal-to-background ratio from 18 to 31 (Figure 1c,d).

### 3.3. Protease Inhibitor Cocktail

Proteases are vital enzymes in *E. coli* and other organisms that break down proteins back into amino acid monomers, which can be used to help meet a cell’s protein-translation demands. Previously reported cell-free systems experienced isotope label scrambling that was reduced with protease inhibitor cocktail [50]. This work quantifies that the protease inhibitor cocktail is compatible with cell-free protein synthesis (Figure S2) and that biosensor signal strength was maintained (Figure S3). Within the context of Δ*glnA* cell extract, the addition of protease inhibitor improves the signal-to-background ratio from 9 to 18 (Figure 1c,d). For Δ*glnA* cell extract treated with MSO, the addition of protease inhibitor improves the signal-to-background ratio from 17 to 31 (Figure 1c,d).

### 3.4. Filtration with Dialysis Membrane

Filtration with a dialysis membrane removes small molecules, such as glutamine, that are present in the cell cytoplasm at the time of lysis. We previously dialyzed cell extract to remove residual free glutamine with the original BL21 Star DE3 strain, but this effort did not increase the signal-to-background ratio, presumably because glutamine synthetase was retained in the cell extract, continuing to produce glutamine [38]. As part of this work, knocking out glutamine synthetase and adding MSO and protease inhibitor reduced the majority of the assay background (Figure 1c,d). Of the remaining assay background, we hypothesized that cytoplasmic glutamine was an appreciable contributing source that could now be addressed with dialysis. Thus, the former hypothesis that dialysis removes residual free glutamine from the extract, which was retested in the context of the knockout Δ*glnA* extract. For the dialyzed Δ*glnA* extract, the assay background was indistinguishable from the instrument background. Thus, this hypothesis that dialysis reduces assay background is correct (Figure 1c,d). Dialysis had the highest signal-to-background ratio of any formulation in this work, but with the critical drawback of a loss of signal strength by more than a factor of 3, which is undesirable for sensor performance (Figure 1c,d).

### 3.5. Combinatorial Insights

Each of the background-reduction strategies (Δ*glnA*, MSO, protease inhibitor, and filtration) contributed to reduced background levels, which improved signal-to-background ratios (Figure 1c,d). However, an increase in signal-to-background ratio was generally inversely proportional to signal strength as shown in Figure 1d. Both a high signal strength and a high signal-to-background ratio are required for an observer to visually distinguish sensor outputs from each other and the background. While the best signal-to-background ratios were observed for dialyzed cell extract, the tradeoff of losing more than 3-fold signal strength makes dialysis relatively unappealing for this biosensor application. For the desired objective of a rapid biosensor, the combination of Δ*glnA*, MSO, and protease inhibitor resulted in the best sensor with a high signal-to-background ratio of 31 and a signal strength of 454 RFU to help promote sensor responses that can be visually distinguished from assay background and other responses, respectively (454 RFU equates to 212 μg/mL sfGFP, which is visible to the naked eye). This work culminates in a rapid, paper-based, colorimetric glutamine assay engineered with the optimal formulation of Δ*glnA*, MSO, and protease inhibitor to balance the tradeoff between signal-to-background ratio and signal strength (Figure 3).

The engineered biosensor with a colorimetric reporter, β-gal/CRPG, demonstrates dramatically enhanced signal resolution compared to our prior work that only used MSO. The assay’s rapid response time is preserved, where saturating concentrations of glutamine produce an assay response after less than 15 min. Importantly, the assay background limitations from prior work have been overcome with the newly engineered formulation. The signal resolution separating signal and background on our prior work was only about 15 min, which has now been extended to 60-plus minutes (Figure 3 and Figure S3). This broad range provides time for intermediate responses to be more clearly distinguished. While clinically relevant serum glutamine concentrations for healthy patients are between approximately 400 μM and 900 μM [24,69], concentrations above 200 μM glutamine are not differentiable within the dynamic range of this sensor formulation. Prior work has demonstrated that dilution with portable pipettes can be used if a sample presumably contains glutamine concentrations that are initially beyond the range of our sensor [39], while other sample types, such as saliva [70], that have glutamine concentrations within our dynamic range would not require dilution. The conditions using just the Δ*glnA* extract (Figure S3a) and using the four combined background-reduction strategies (Figure S3c) also functioned as biosensors, although with a tradeoff of reduced dynamic range relative to the optimal conditions reported in Figure 3 (Figure S3). The limit of detection of this assay in its current form is between 2 and 20 μM glutamine, as shown in Figure 3b. While efforts to tune the limit of detection, selectivity, and other assay parameters are continuing, the herein reported work to engineer the sensor for enhanced signal resolution overcomes a critical limitation of the prior assay. Notably, prior work has conducted selectivity studies of the glutamine sensor and related amino acids with encouraging results [38].

While demonstrating the sensor performance with human samples is beyond the scope of this work to engineer the sensor for enhanced signal resolution, our prior work has demonstrated that cell-free sensors are compatible with human serum, blood, saliva, and urine [38,39,71], which is encouraging for the potential success of future work in clinical validation.

## 4. Conclusions

Numerous preclinical and clinical studies have established great potential for cancer treatments targeting glutamine dependence where glutamine itself is a valuable biomarker. Unfortunately, portable treatment-monitoring tests are unavailable to cancer patients, thus limiting patient access to life-saving therapies. To address the need for a low-cost, portable glutamine diagnostic, this work engineers a low-cost, paper-based, colorimetric glutamine sensor to overcome previous assay background limitations using genetic engineering, chemical inhibitors, and process optimization. This work quantifies a tradeoff between signal-to-background ratio and signal strength, revealing an optimal biosensor formulation. The culmination of this work is the use of this optimal formulation to develop a shelf-stable, paper-based, colorimetric glutamine test with a high signal strength and high signal-to-background ratio.

The optimized, low-cost, portable biosensor marks important progress toward safer and more effective cancer treatments involving glutamine depletion treatment. Other biosensor assay-development efforts could also benefit from these findings. The assay background-mitigation strategies presented in this work could be applied to other biosensors targeting other important analytes.

## Data Availability

The data presented in this study are available within the article and supplementary information.

## Supplementary Materials

The following supporting information can be downloaded at https://www.mdpi.com/article/10.3390/s24103073/s1, Figure S1: BL21 Star DE3 Δ*lac* Δ*glnA* glutamine auxotroph phenotype; Figure S2: Protease inhibitor toleration of cell-free protein synthesis; Figure S3: Visual and quantitative paper-based cell-free glutamine biosensor results; Figure S4: A superimposed quantitative comparison of all tested conditions reported in Figure S3; Figure S5: An original uncropped photograph showing the glutamine biosensor; Figure S6: DNA sequences used in this work.
