# Supplementary material for: Engineering the Signal Resolution of a Paper-Based Cell-Free Glutamine Biosensor with Genetic Engineering, Metabolic Engineering, and Process Optimization
Source: Sensors (Basel). 2024 May 12;24(10):3073. doi: 10.3390/s24103073 (PMC11124800; doi:10.3390/s24103073)
Supplement: Supplementary file 1 [file sensors-24-03073-s001.zip › sensors-2965486-supplementary.pdf]

sensors

Article: Supplementary Information

Engineering the Signal Resolution of a Paper-based Cell-free Glutamine Biosensor with Genetic Engineering, Metabolic Engineering, and Process Optimization

Tyler J. Free [1], Joseph P. Talley [1], Chad D. Hyer [1], Catherine J. Miller [1], Joel S. Griffitts [2], and Bradley C. Bundy [1,*]

[1] Department of Chemical Engineering, Brigham Young University, Provo, UT 84602, USA
[2] Department of Microbiology and Molecular Biology, Brigham Young University, Provo, UT 84602, USA
* Correspondence: bundy@byu.edu

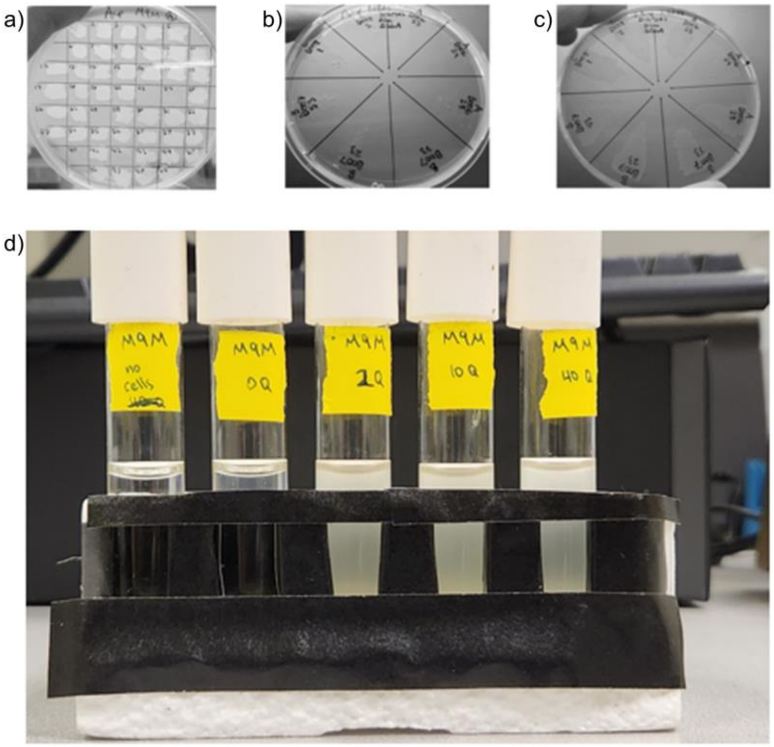

Figure S1: BL21 Star DE3 Δ*lac* Δ*glnA* glutamine auxotroph phenotype. To isolate the auxotrophic colonies after the MAGE mutagenesis protocol, individual colonies were isolated on LB culture plates. Colonies were then numbered and patched onto squares of a minimal media plate (**a**). The colonies corresponding to the cell patches that failed to grow were patched again on minimal media plates whereon they did not grow (**b**), and patched again on standard LB plates whereon they did grow for further propagation (**c**). (**d**) An original uncropped photograph for Figure 2 showing the test tube rack apparatus used for photographing 5 mL liquid *E. coli* cultures in test tubes, which were incubated at 37 °C and 280 RPM and briefly placed in the test tube rack for photographing at indicated time points.

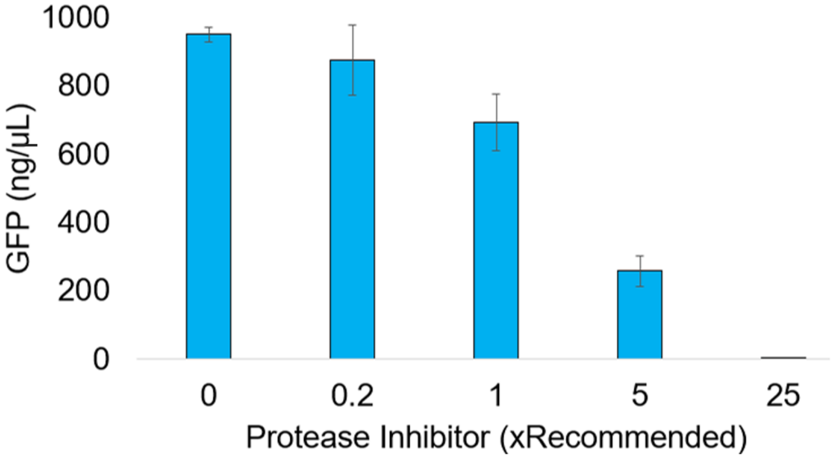

Figure S2: Protease inhibitor toleration of cell-free protein synthesis. The cell-free sfGFP production yields in the presence of varying concentrations of protease inhibitor cocktail, given as normalized multipliers relative to the concentration recommended by the manufacturer for cell lysate.

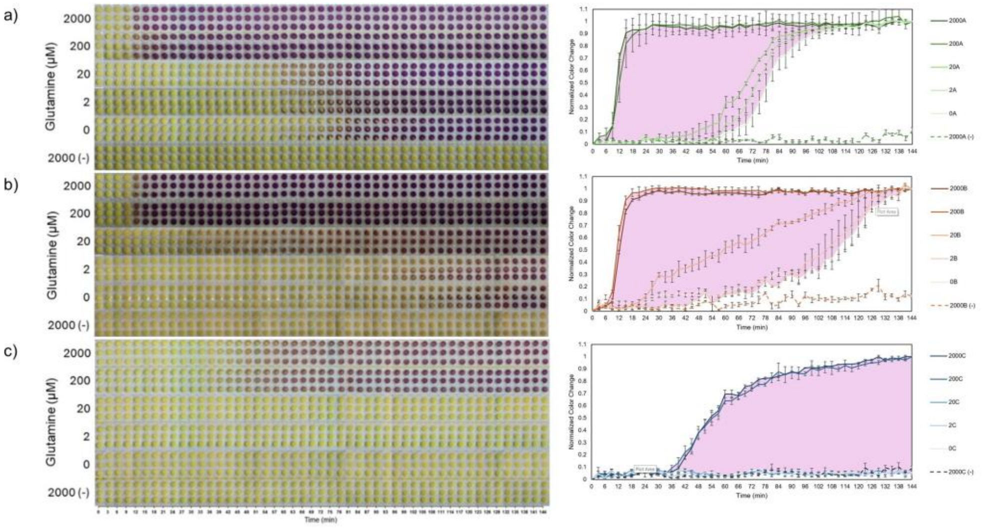

Figure S3: Visual and quantitative paper-based, cell-free glutamine biosensor results. Based on the signal-to-background ratio and signal strength tradeoff shown in Figure 1d, the following three biosensor formulations were engineered into a lyophilized, colorimetric, paper-based format and tested with different glutamine concentrations. (**a**) Δ*glnA* knockout extract with no additives. (**b**) Δ*glnA* knockout extract with MSO and protease inhibitor added. (**c**) Filtered Δ*glnA* knockout extract with MSO and protease inhibitor. Glutamine concentrations (in µM) are indicated on the axis or legend. The quantitative sensor responses are shown on the normalized plots to the right, where the region between the maximum signal and assay background is shaded.

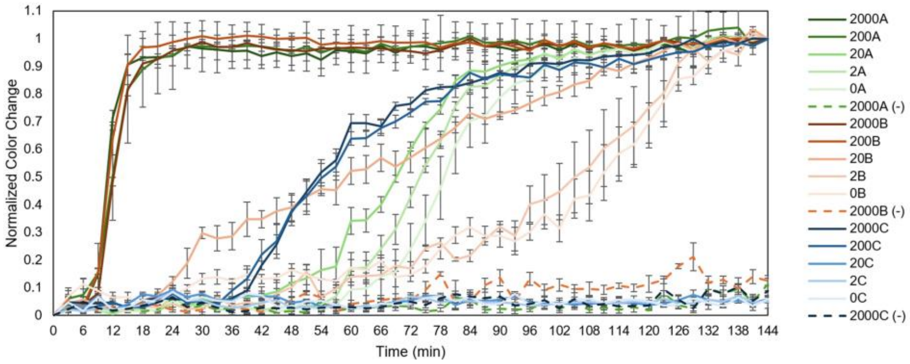

Figure S4: A superimposed quantitative comparison of all tested conditions reported in Figure S3.

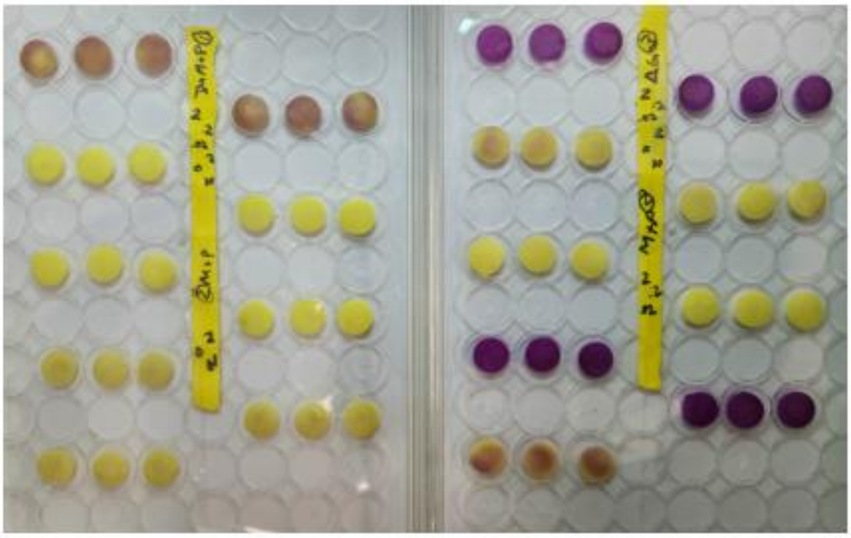

Figure S5: An original uncropped photograph showing the glutamine biosensor. Repeat images of the tests were captured every 3 minutes. To create Figure 2 and Figure S3, these images were cropped individually for each set of triplicates using Adobe Lightroom, and the images were oriented in rows to show the time-dependent glutamine outcome for each concentration tested.

Figure S6: DNA sequences used in this work.

***lacZ* Single Stranded Oligo (26 bp deletion)**

GCTGCGCTGGAGTGACGGCAGTTATCTGGAAGATCAGGATATGTGTCTCGTT-GCTGCATAAACCGACTACACAAATCAGCGATTTCCATG

***glnA* Single Stranded Oligo (10 bp deletion)**

CACGTCACTATCCCTGCTCATCAGGTGAATGCTGAATTCTCAAAATGTTTGAC-GGCTCCTCGATTGGCGGCTGGAAAGGC

MAGE plasmid pJG1219

GGTACCAAATTCCAGAAAAGAGGCCTCCCGAAAGGGGGGCCTTTTTTCGTTTTG
GTCCAATGGCGGCGCGCCATCGAATGGTGCAAAACCTTTCGCGGTATGGCATGA
TAGCGCCCGGAAGAGAGTCAATTCAGGGTGGTGAATATGAGCCCGAAACGTCGT
ACCCAGGCAGAACGTGCAATGGAAACCCAGGGTAAACTGATTGCAGCAGCACT
GGGTGTTCTGCGTGAAAAAGGTTATGCAGGTTTTCGTATTGCAGATGTTCCGGGT
GCAGCCGGTGTTAGCCGTGGTGCACAGAGCCATCATTTTCCGACCAAACTGGAA
CTGCTGCTGGCAACCTTTGAATGGCTGTATGAGCAGATTACCGAACGTAGCCGTG
CACGTCTGGCAAAACTGAAACCGGAAGATGATGTTATTCAGCAGATGCTGGATG
ATGCAGCAGAATTTTTTCTGGATGATGATTTTAGCATCGGCCTGGATCTGATTGTT
GCAGCAGATCGTGATCCGGCACTGCGTGAAGGTATTCAGCGTACCGTTGAACGT
AATCGTTTTGTTGTTGAAGATATGTGGCTGGGTGTGCTGGTGAGCCGTGGTCTGA
GCCGTGATGATGCCGAAGATATTCTGTGGCTGATTTTTAACAGCGTTCGTGGTCT
GGTAGTTCGTAGCCTGTGGCAGAAAGATAAAGAACGTTTTGAACGTGTGCGTAA
TAGCACCCTGGAAATTGCACGTGAACGTTATGCAAAATTCAAACGTTGATAAGG
ATCCAATTATTGAAGGCCTCCCTAACGGGGGGCCTTTTTTTGTTTCTGGTCTCCCG
CTTAACGATCGTTGGCTGAACAAACAGACAATCTGGTCTGTTTGTATTATGGAAA
ATTTTTCTGTATAATAGATTCAACAAACAGACAATCTGGTCTGTTTGTATTATAGC
TGTCTAGACTTTAGGAGGTATATATGAGTACTGCACTCGCAACGCTGGCTGGGA
AGCTGGCTGAACGTGTCGGCATGGATTCTGTCGACCCACAGGAACTGATCACCA
CTCTTCGCCAGACGGCATTTAAAGGTGATGCCAGCGATGCGCAGTTCATCGCATT
ACTGATCGTTGCCAACCAGTACGGCCTTAATCCGTGGACGAAAGAAATTTACGC
CTTTCCTGATAAGCAGAATGGCATCGTTCCGGTGGTGGGCGTTGATGGCTGGTCC
CGCATCATCAATGAAAACCAGCAGTTTGATGGCATGGACTTTGAGCAGGACAAT
GAATCCTGTACATGCCGGATTTACCGCAAGGACCGTAATCATCCGATCTGCGTTA
CCGAATGGATGGATGAATGCCGCCGCGAACCATTCAAAACTCGCGAAGGCAGA
GAAATCACGGGGCCGTGGCAGTCGCATCCCAAACGGATGTTACGTCATAAAGCC
ATGATTCAGTGTGCCCGTCTGGCCTTCGGATTTGCTGGTATCTATGACAAGGATG
AAGCCGAGCGCATTGTCGAAAATACTGCATACACTGCAGAACGTCAGCCGGAA
CGCGACATCACTCCGGTTAACGATGAAACCATGCAGGAGATTAACACTCTGCTG
ATCGCCCTGGATAAAACATGGGATGACGACTTATTGCCGCTCTGTTCCCAGATAT
TTCGCCGCGACATTCGTGCATCGTCAGAACTGACACAGGCCGAAGCAGTAAAAG
CTCTTGGATTCCTGAAACAGAAAGCCGCAGAGCAGAAGGTGGCAGCATGAAGC
TTTGAGAGAGGAGGATTACAAAATGCCAATTCAGGTCTTACCGCCACAACTGGC
GAACCAGATTGCCGCAGGTGAGGTGGTCGAGCGACCTGCGTCGGTAGTCAAAG
AACTAGTGAAAAACAGCCTCGATGCAGGTGCGACGCGTATCGATATTGATATCG
AACGCGGTGGGGCGAAACTTATCCGCATTCGTGATAACGGCTGCGGTATCAAAA
AAGATGAGCTGGCGCTGGCGCTGGCTCGTCATGCCACCAGTAAAATCGCCTCTC
TGGACGATCTCGAAGCCATTATCAGCCTGGGCTTTCGCGGTGAGGCGCTGGCGA
GTATCAGTTCGGTTTCCCGCCTGACGCTCACTTCACGCACCGCAGAACAGCAGG
AAGCCTGGCAGGCCTATGCCGAAGGGCGCGATATGAACGTGACGGTAAAACCG
GCGGCGCATCCTGTGGGGACGACGCTGGAGGTGCTGGATCTGTTCTACAACACC

CCGGCGCGGCGCAAATTCCTGCGCACCGAGAAAACCGAATTTAACCACATTGAT
GAGATCATCCGCCGCATTGCGCTGGCGCGTTTCGACGTCACGATCAACCTGTCGC
ATAACGGTAAAATTGTGCGTCAGTACCGCGCAGTGCCGGAAGGCGGGCAAAAA
GAACGGCGCTTAGGCGCGATTTGCGGCACCGCTTTTCTTGAACAAGCGCTGGCG
ATTGAATGGCAACACGGCGATCTCACGCTACGCGGCTGGGTGGCCGATCCAAAT
CACACCACGCCCGCACTGGCAGAAATTCAGTATTGCTACGTGAACGGTCGCATG
ATGCGCGATCGCCTGATCAATCACGCGATCCGCCAGGCCTGCGAAGACAAACTG
GGGGCCGATCAGCAACCGGCATTTGTGTTGTATCTGGAGATCGACCCACATCAG
GTGGACGTCAACGTGCACCCCGCCAAACACGAAGTGCGTTTCCATCAGTCGCGT
CTGGTGCATGATTTTATCTATCAGGGCGTGCTGAGCGTGCTACAACAGCAACTGG
AAACGCCGCTACCGCTGGACGATGAACCCCAACCTGCACCGCGTTCCATTCCGG
AAAACCGCGTGGCGGCGGGGCGCAATCACTTTGCAGAACCGGCAGCTCGTGAG
CCGGTAGCTCCGCGCTACACTCCTGCGCCAGCATCAGGCAGTCGTCCGGCTGCC
CCCTGGCCGAATGCGCAGCCAGGCTACCAGAAACAGCAAGGTGAAGTGTATCG
CCAGCTTTTGCAAACGCCCGCGCCGATGCAAAAATTAAAAGCGCCGGAACCGC
AGGAACCTGCACTTGCGGCGAACAGTCAGAGTTTTGGTCGGGTACTGACTATCG
TCCATTCCGACTGTGCGTTGCTGGAGCGCGACGGCAACATTTCACTTTTATCCTTG
CCAGTGGCAGAACGTTGGCTGCGTCAGGCACAATTGACGCCGGGTGAAGCGCCC
GTTTGCGCCCAGCCGCTGCTGATTCCGTTGCGGCTAAAAGTTTCTGCCGAAGAAA
AATCGGCATTAGAAAAAGCGCAGTCTGCCCTGGCGGAATTGGGTATTGATTTCC
AGTCAGATGCACAGCATGTGACCATCAGGGCAGTGCCTTTACCCTTACGCCAAC
AAAATTTACAAATCTTGATTCCTGAACTGATAGGCTACCTGGCGAAGCAGTCCGT
ATTCGAACCTGGCAATATTGCGCAGTGGATTGCACGAAATCTGATGAGCGAACA
TGCGCAGTGGTCAATGGCACAGGCCATAACCCTGCTGGCGGACGTGGAACGGTT
ATGTCCGCAACTTGTGAAAACGCCGCCGGGTGGTCTGTTACAATCTGTTGATTTA
CATCCGGCGATAAAAGCCCTGAAAGATGAGTGAAATGGCGATGAGCATCCTCA
CGAGCTCTTAGGCTAGCTGACACGCTTAAGGCCTCCCAATCGGGAGGCCTTTTTT
TGTTCCACTGAGCGTCAGACCCCTTAATAAGATGATCTTCTTGAGATCGTTTTGGT
CTGCGCGTAATCTCTTGCTCTGAAAACGAAAAAACCGCCTTGCAGGGCGGTTTTT
CGAAGGTTCTCTGAGCTACCAACTCTTTGAACCGAGGTAACTGGCTTGGAGGAG
CGCAGTCACCAAAACTTGTCCTTTCAGTTTAGCCTTAACCGGCGCATGACTTCAA
GACTAACTCCTCTAAATCAATTACCAGTGGCTGCTGCCAGTGGTGCTTTTGCATG
TCTTTCCGGGTTGGACTCAAGACGATAGTTACCGGATAAGGCGCAGCGGTCGGA
CTGAACGGGGGGTTCGTGCATACAGTCCAGCTTGGAGCGAACTGCCTACCCGGA
ACTGAGTGTCAGGCGTGGAATGAGACAAACGCGGCCATAACAGCGGAATGACA
CCGGTAAACCGAAAGGCAGGAACAGGAGAGCGCACGAGGGAGCCGCCAGGGG
GAAACGCCTGGTATCTTTATAGTCCTGTCGGGTTTCGCCACCACTGATTTGAGCG
TCAGATTTCGTGATGCTTGTCAGGGGGGCGGAGCCTATGGAAAAACGGCTTTGC
CGCGGCCCTCTCACTTCCCTGTTAAGTATCTTCCTGGCATCTTCCAGGAAATCTCC
GCCCCGTTCGTAAGCCATTTCCGCTCGCCGCAGTCGAGCAATTGCCAGCTGGAGC
GCCCTCTGGTAAGGTTGGGAAGCTAGACTTTAGGAGGTATACATATGAGTATTCA
ACATTTCCGTGTCGCCCTTATTCCCTTTTTTGCGGCATTTTGCCTTCCTGTTTTTGCT

CACCCAGAAACGCTGGTGAAAGTAAAAGATGCTGAAGATCAGTTGGGTGCACG
AGTGGGTTACATCGAACTGGATCTCAACAGCGGTAAGATCCTTGAGAGTTTTCGC
CCCGAAGAACGTTTTCCAATGATGAGCACTTTTAAAGTTCTGCTATGTGGCGCGG
TATTATCCCGTATTGACGCCGGGCAAGAGCAACTCGGTCGCCGCATACACTATTC
TCAGAATGACTTGGTTGAGTACTCACCAGTCACAGAAAAGCATCTTACGGATGG
CATGACAGTAAGAGAATTATGCAGTGCTGCCATAACCATGAGTGATAACACTGC
GGCCAACTTACTTCTGACAACGATCGGAGGACCGAAGGAGCTAACCGCTTTTTT
GCACAACATGGGGGATCATGTAACTCGCCTTGATCGTTGGGAACCGGAGCTGAA
TGAAGCCATACCAAACGACGAGCGTGACACCACGATGCCTGTAGCAATGGCAA
CAACGTTGCGCAAACTATTAACTGGCGAACTACTTACTCTAGCTTCCCGGCAACA
ATTAATAGACTGGATGGAGGCGGATAAAGTTGCAGGACCACTTCTGCGCTCGGC
CCTTCCGGCTGGCTGGTTTATTGCTGATAAATCTGGAGCCGGTGAGCGTGGGTCT
CGCGGTATCATTGCAGCACTGGGGCCAGATGGTAAGCCCTCCCGTATCGTAGTTA
TCTACACGACGGGGAGTCAGGCAACTATGGATGAACGAAATAGACAGATCGCT
GAGATAGGTGCCTCACTGATTAAGCATTGGTAAGAATTGACCCTCGAAACCGGA
TGCACC

Disclaimer/Publisher's Note: The statements, opinions and data contained in all publications are solely those of the individual author(s) and contributor(s) and not of MDPI and/or the editor(s). MDPI and/or the editor(s) disclaim responsibility for any injury to people or property resulting from any ideas, methods, instructions or products referred to in the content.
